# Seasonal Influenza Vaccine Allocation in the Canadian Population during a Pandemic

**DOI:** 10.1371/currents.RRN1143

**Published:** 2009-12-11

**Authors:** Ashleigh Tuite, David N. Fisman, Jeffrey C. Kwong, Amy Greer

**Affiliations:** ^*^Dalla Lana School of Public Health, University of Toronto; ^†^Institute for Clinical Evaluative Sciences, Toronto, Ontario, Canada and ^‡^Public Health Agency of Canada

## Abstract

Introduction: Emerging data suggest that receipt of the seasonal influenza vaccine may be associated with an enhanced risk of infection with pandemic (H1N1) 2009 (pH1N1). We sought to evaluate different seasonal vaccination strategies during a pandemic in the presence of varying levels of pH1N1 infection risk following seasonal influenza vaccine receipt.

Methods: We developed a deterministic, age-structured compartmental model of influenza transmission in the presence of two circulating strains (pH1N1 and seasonal). We examined the effect of different seasonal vaccination strategies on total influenza-attributable mortality in the Canadian population for the 2009-2010 influenza season.

Results: Seasonal vaccination strategies that focused on individuals aged ≥65 or delayed seasonal vaccine delivery until January tended to minimize mortality. In the presence of low levels (<2%) of co-circulating seasonal influenza, mortality estimates were sensitive to the seasonal vaccine-associated relative risk (RR), with small increases in RR resulting in enhanced mortality compared to the no seasonal vaccination option. Timing of the peak of pH1N1 activity and the amount of circulating seasonal influenza modified the impact of enhanced risk on total mortality.

Discussion: In the presence of uncertainty surrounding enhanced risk of pH1N1 acquisition with seasonal vaccine receipt, delaying seasonal vaccine delivery or restricting vaccine to individuals aged ≥65 may reduce overall influenza-attributable mortality in the Canadian population.

## **Introduction**

 As the world confronts the first influenza pandemic of the 21^st^ century, countries need to make decisions about disease containment and control in the face of uncertainty.  Influenza immunization is an important component of pandemic preparedness planning and is an effective preventive measure for reducing influenza-related morbidity and mortality [1].  Development of a vaccine against pandemic (H1N1) 2009 (pH1N1) began in the early phases of the epidemic.  In the northern hemisphere, seasonal influenza vaccine production was already completed for the 2009-2010 influenza season. As a result, questions have arisen regarding the need to administer both vaccines and the logistical complexities surrounding the implementation of two population-wide vaccination programs in a single influenza season. 

During a pandemic, co-circulation of seasonal influenza strains is typically reduced due to strain replacement by the pandemic strain [2];  the experience in the southern hemisphere and other recent data suggest that this may also be observed in the upcoming influenza season [3] [4] [5].  In such a situation, the importance of administering seasonal influenza vaccine is decreased. 

Canada’s influenza season typically occurs at some point between November and April, with an estimated 10 to 25% of the population reporting influenza-like illness each year [1].  Seasonal influenza vaccination coverage varies across provinces and age-groups, with highest uptake in the elderly and individuals with underlying medical conditions for which influenza immunization is recommended.  Emerging data from three provinces in Canada (British Columbia, Ontario and Quebec) indicate that vaccination against seasonal influenza may enhance the risk of acquiring pH1N1 infection [6].  The biological basis of this observation is unclear, and the results have not been replicated in other jurisdictions.  We sought to investigate how the potential for enhanced risk might modify seasonal vaccine recommendations for Canada.  In particular, at what level of enhanced risk of infection with pH1N1 would the reduced seasonal influenza-attributable morbidity and mortality conferred by the seasonal vaccine be negated by increased infection (and resultant morbidity and mortality) due to pH1N1?  To address this question, we developed an age-structured mathematical model with two circulating influenza strains to describe influenza transmission in the upcoming influenza season.  We used this model to evaluate the impact of different seasonal vaccination strategies on overall influenza-attributable mortality.  We also considered the effect of different proportions of seasonal influenza among circulating strains and different relative risks of pH1N1 infection associated with receipt of the seasonal vaccine.

## **Methods**

### Model structure

We developed a deterministic, age-structured compartmental model of influenza transmission with two circulating strains (seasonal and pH1N1) in the Canadian population (see **Figure 1** for overall structure).  Although seasonal influenza is characterized by a mixture of influenza A and B subtypes, we treated seasonal influenza as a single strain to reduce model complexity.   The model ran from mid-April, 2009 (the date of the first identified cases of pH1N1 in Canada) to June 30, 2010, representing a single influenza season.  As a result, we did not consider waning immunity following infection or vaccination, migration into or out of the population, or population aging.  

**Figure:**
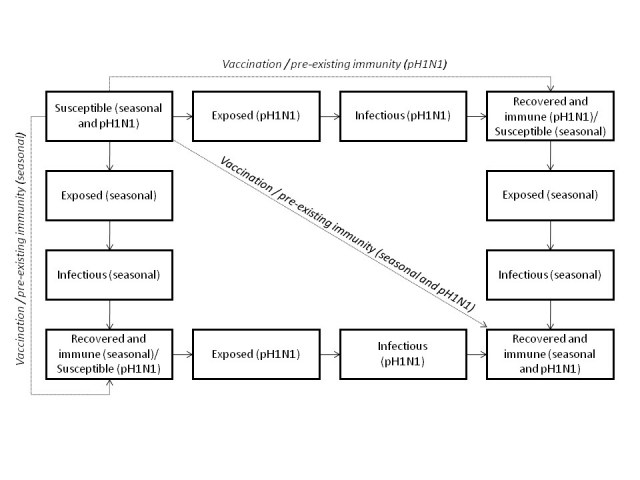
**Figure 1.  **Outline of model structure, showing population flows between epidemiological compartments.  Each compartment is further stratified by age category.  pH1N1 and seasonal refer to pandemic (H1N1) 2009 and seasonal influenza, respectively.

### Disease transmission

The population was divided into four compartments representing different disease states: susceptible (*S*), exposed (*E*; i.e., infected but not infectious), infectious (*I*), and recovered (*R*).  Transmission of infection occurred through contact between susceptible and infectious individuals.  Individuals transitioned between these compartments for either of the influenza strains.  Individuals who recovered from infection with one strain remained susceptible to the second strain.  We did not consider co-infection.  We assumed that 40% of infections were asymptomatic [7], but did not consider differential transmission in symptomatic versus asymptomatic cases.   

### Age structure and mixing patterns

To explore how seasonal vaccination of different age groups would impact overall influenza-attributable mortality and to enable the representation of more realistic contact patterns within and between age groups, we introduced age stratification.  The Canadian population was divided into seven age classes with the following cutoffs:  0-4, 5-13, 14-17, 18-22, 23-52, 53-64 and ≥65.  Demographic information was obtained from 2006 Canadian census data [8].  We included the 53-64 year old age category in order to model the decreased susceptibility to infection with pH1N1 observed in persons born prior to 1957 [9] [10] [11].  Population mixing within and between age strata was based on a population-based prospective study of mixing patterns in eight European countries [12].  

### Pre-existing immunity

To reflect the presence of immunity to circulating influenza strains in the population due to previous exposure, resistance to either seasonal influenza, pH1N1, or both strains was modeled by removing individuals from the susceptible population at time zero.  These individuals were moved to the appropriate resistant pools, and were still capable of being infected with the influenza strain to which they did not have immunity if they were not already resistant to both strains (due to either pre-existing immunity to both or a combination of previous exposure to one strain and immunization against the other strain).  Since it is not possible to distinguish individuals with pre-existing exposure to the circulating strains, we assumed that they received the same vaccination coverage as the susceptible population (i.e. there was no way to preferentially immunize the truly susceptible population).  

### Disease natural history and model parameterization

Model parameters for seasonal influenza were derived from the published literature[13] [14] [15] [16] (**Table 1**).  Epidemiological parameters for pH1N1 were based on the currently available case data from the province of Ontario [16].  The proportion of the population resistant to infection with seasonal or pH1N1 influenza due to prior exposure was estimated during model calibration.  Age-specific case fatality rates (CFR) for seasonal influenza were based on previously published data [14].  Age-specific CFR for pH1N1 were calculated using data from Ontario’s Integrated Public Health Information System (iPHIS), which collected information on all laboratory-confirmed cases of pH1N1 in the province reported between April 13 and June 21, 2009.  To account for expected under-ascertainment of less severe cases, we multiplied the denominator (total cases) by a factor of ten when calculating CFR [17].

**Table 1. **Model parameter values. 

**Table:** 

** Variable **	** Age group **	** Seasonal **	** pH1N1**	** Source**
Total population size	0-4 5-13 14-17 18-22 23-52 53-64 ≥65	1,690,550 3,456,700 1,746,045 2,088,775 13,729,530 4,566,055 4,335,250	1,690,550 3,456,700 1,746,045 2,088,775 13,729,530 4,566,055 4,335,250	Statistics Canada, 2007 [8]
Latent period (days)	All	2.1	3.5	Chowell et al., 2008 [13]; Tuite et al., 2009 [16]; Model calibration
Duration of infectiousness (days)	All	4.8	2.5	Chowell et al., 2008 [13][13]; Tuite et al., 2009 [16]; Model calibration
Reproductive number	All	1.3- 1.4	1.3	Model calibration
Proportion of population with pre-existing immunity	0-4 5-17 18-52 ≥53	0 0.15 0.3 0.3	0 0 0 0.5	Model calibration
Vaccine effectiveness	0-64 ≥65	0.7 0.5	0.7 0.5	CDC, 2008 [18]
Vaccination coverage	0-4 5-13 14-17 18-22 23-52 53-64 ≥65	0.27 0.18 0.19 0.19 0.21 0.36 0.69	0.26 0.30 0.31 0.29 0.29 0.47 0.75	Kwong et al., 2008[15]; Moran et al., 2009 [19]
Case fatality rate	0-4 5-17 18-52 53-64 ≥65	0.00004 0.00001 0.00009 0.0013 0.012	0 0.000064 0.00025 0.0039 0.0039	Molinari et al., 2007 [14]; Tuite et al., 2009 [16]

#### Vaccination scenarios 

Vaccination with a single dose of seasonal or pH1N1 vaccine was modeled by removing a select number of individuals from all model compartments except ‘infectious’ at a given point in time.  Vaccination was considered to occur simultaneously across the population, with a two-week delay between vaccine administration and development of a protective immune response.  The fraction of the vaccinated population that became resistant was based on age-specific vaccine effectiveness estimates; for a given age group, with a vaccine effectiveness *VE* and coverage *C*, the proportion removed from the susceptible pool was VE*C.  We assumed that this group was fully protected against infection, with the remaining fraction VE*(1-*C*) receiving no protection.  Although this does not reflect the true situation, where most vaccinated individuals will experience some degree of protection, this approach has been used previously and has been demonstrated to provide a reasonable model of partial efficacy [20].  

For all scenarios, we assumed that pH1N1 vaccination occurred in mid-November, with the peak of pH1N1 activity occurring in either mid-November (basic reproductive number (R0) decreased from 1.3 to 1.15 from July to September) or mid-January (R0 decreased from 1.3 to 1 from July to October).  To model expected pH1N1 vaccine uptake, we used vaccination coverage data for the province of Ontario [15] [19] which operates a universal influenza immunization program that provides influenza vaccine free of charge to the entire population aged six months or older.

We considered four seasonal vaccination strategies:   

1. No seasonal vaccination
2. Seasonal vaccination in early October
3. Seasonal vaccination in early January
4. Seasonal vaccination of individuals ≥65 in early October followed by vaccination of individuals <65 in early January

For scenarios (2) and (3) we evaluated both vaccination of the entire population and vaccination of the ≥65 age group only. Rates of seasonal vaccine uptake were based on Canada-wide estimates of seasonal vaccination coverage [15] [19].  

We modeled changes in the relative risk of infection with pH1N1 following receipt of seasonal influenza vaccine in individuals who received the seasonal vaccine and had not been previously infected with pH1N1 by multiplying their risk of pH1N1 exposure by the vaccine-associated relative risk (ranging from 0.9 to 2.0).  We assumed that receipt of the seasonal vaccine in seasonal influenza-susceptible individuals was the only route of enhancing the risk of pH1N1 infection (i.e., individuals who were naturally exposed to seasonal influenza were not at enhanced risk if they received the vaccine following natural infection).  

We assumed that seasonal influenza was first introduced into the population at the end of August.  To simulate changes in the proportion of circulating seasonal influenza relative to pH1N1, we used different values of the basic reproductive number (R0) for seasonal influenza (ranging from 1.3 (1.8% of circulating strains) to 1.4 (16% of circulating strains), and dropped R0 to 0.9 in May, 2010).   The amount of seasonal influenza in circulation was calculated as the proportion of seasonal influenza among the expected number of infections with both strains in the absence of vaccination.  

The outcome of interest was total influenza-attributable mortality.  We evaluated the effectiveness of different vaccination strategies by calculating the percent change in mortality relative to predicted mortality in the absence of seasonal vaccination campaigns.  

#### Model calibration

To verify that the seasonal influenza transmission model was reproducing typical patterns of seasonal epidemics, we compared model outputs with data on influenza-attributable excess mortality.  Time-series data were available for three years, covering three influenza seasons. Mortality data represented all-cause mortality above expected baseline mortality in the absence of influenza and included data from all provinces but not the three territories (representing 0.32% of the Canadian population) [15].  Data were also available on self-reported vaccination rates [15].  Parameters were varied (R0, duration of infectiousness, latent period, proportion resistant to infection) to minimize the sum-of-squares difference between model-predicted and reported deaths.  For pH1N1, the model was calibrated to fit the initial epidemic curve observed in Ontario. Data for cases with a reported exposure date between April 16 and May 25, 2009 were obtained from iPHIS.  Travel history data, including illness on return to Mexico, were used to model the observed multiple introductions of pH1N1 into the population early on in the pandemic.

## **Results**

### Mortality in the absence of seasonal influenza vaccination

We assessed total mortality when seasonal influenza represented an increasing percentage of total circulating strains, with pH1N1 comprising the remaining influenza in circulation for the 2009-2010 season (**Figure 2**). In the absence of seasonal influenza vaccination programs and with pH1N1 vaccination occurring in mid-November, total influenza-attributable mortality was predicted to increase as the proportion of seasonal influenza increased.  Overall mortality was greater for the November pH1N1 epidemic peak than the January peak, since we assumed that pH1N1 vaccine was administered in mid-November, reducing its effectiveness as a mitigation strategy when pH1N1 activity was already high.

**Figure:**
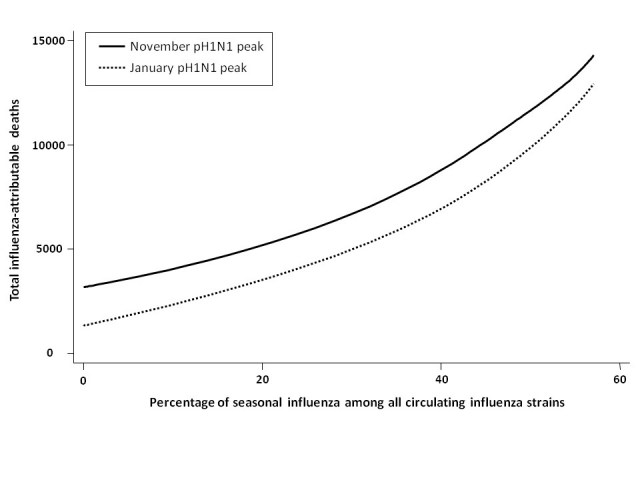
**Figure 2.  **Total model-predicted influenza-attributable mortality in the presence of differing amounts of seasonal influenza relative to pH1N1.  Mortality was estimated in the absence of seasonal vaccination, with pH1N1 vaccination occurring in mid-November and the peak of pH1N1 activity occurring in either November or January.

### Effect of seasonal vaccine-associated relative risk on overall mortality 

We assessed total influenza-attributable mortality when seasonal influenza accounted for 1.8% (low), 6.2% (moderate), or 16.2% (high) of total circulating strains, with pH1N1 representing the remaining circulating influenza strain.  These fractions correspond to R0 values of 1.3, 1.35, and 1.4 for seasonal influenza, respectively.  We compared total model-predicted influenza-attributable mortality in the absence of seasonal vaccination to that predicted to occur if seasonal vaccination campaigns occur as usual in early October, assuming a November pH1N1 epidemic peak (**Figure 3**).   

At low levels of circulating seasonal influenza, when vaccine was distributed to all age groups, the total number of deaths was higher than in the absence of vaccination when the RR of pH1N1 infection associated with vaccine receipt exceeded 1.1.  At moderate levels of circulating seasonal influenza, this effect was observed at a higher RR (>1.4).  With high levels of circulating seasonal influenza, seasonal vaccination always reduced total mortality.  In the presence of high circulating levels of seasonal influenza, reduced mortality with seasonal vaccination was observed for all subsequent scenarios, and so these results are not shown graphically for the remaining simulations.

Administering seasonal vaccine exclusively to those aged 65 and over attenuated the relationship between increasing RR and mortality.  However, at the lowest level of circulating seasonal influenza, the no seasonal vaccination strategy was preferred once the vaccine-associated RR surpassed 1.2.  

When we repeated the analysis assuming a January pH1N1 epidemic peak, a higher vaccine-associated RR was tolerated before the no vaccination approach was preferred (as pH1N1 was attenuated by pandemic vaccine), and this effect was more marked when using the elderly-only strategy for seasonal vaccination.

**Figure:**
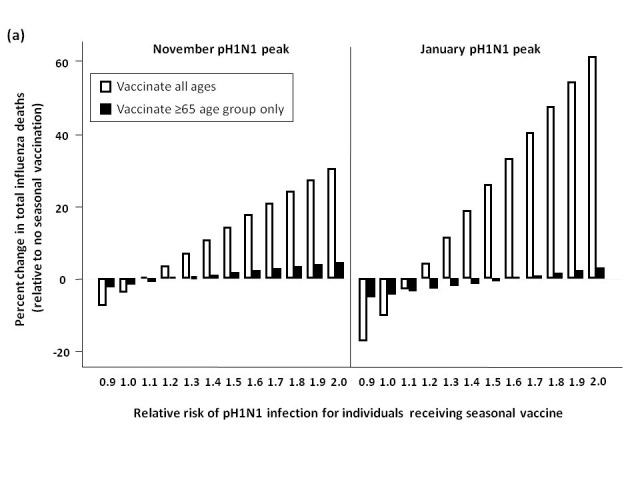

**Figure:**
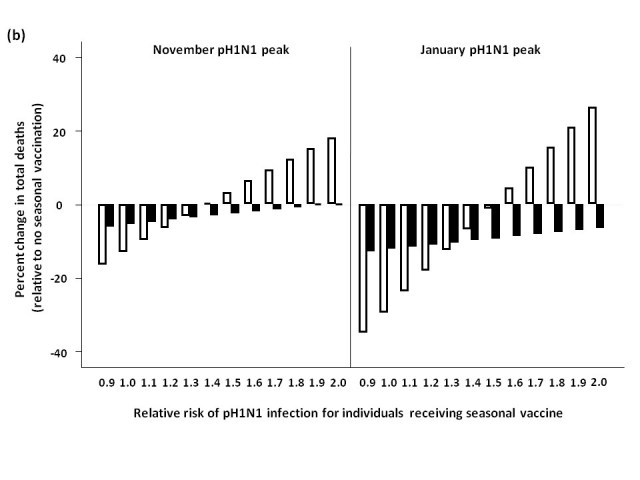

**Figure:**
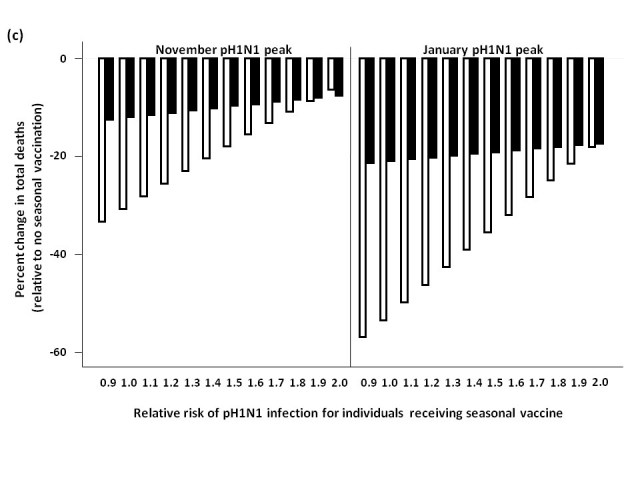
**Figure 3.**  Total predicted change in influenza-attributable mortality, in the presence of (a) low (1.8%), (b) moderate (6%), and (c) high (16%) levels of circulating seasonal influenza.  The effectiveness of administering seasonal vaccine to all age groups or the ≥65 age group in October was evaluated assuming a pH1N1 epidemic peak in November, 2009 or January, 2010, with pH1N1 vaccination occurring in mid-November.  Percent reduction or increase in mortality was calculated relative to predicted mortality in the absence of seasonal vaccine administration.  Vaccination coverage for seasonal and pH1N1 influenza is described in Table 1.

### Seasonal vaccine delay

When pH1N1 activity peaked in November, delaying seasonal vaccine delivery to early January was an effective strategy for minimizing total mortality, compared to administering vaccine in October (**Figure 4**).  For low levels of circulating seasonal influenza, vaccinating all age groups against seasonal strains was less effective than an elderly-focused approach, where mortality was reduced compared to no seasonal vaccination regardless of the vaccine-associated RR; however, the absolute number of deaths prevented in all scenarios was relatively small in the national context (<40 deaths) compared to non-use of seasonal vaccination.

Similarly, for a January pH1N1 epidemic peak and low seasonal strain circulation, delaying seasonal vaccine resulted in a higher RR being tolerated before seasonal vaccination had a negative effect on mortality when vaccinating all age groups.  Vaccinating the ≥65 age group resulted in lower mortality at all levels of RR, compared to no vaccination.  

For both the November and January pH1N1 peaks, in the presence of moderate or high levels of circulating seasonal influenza, a population-wide or elderly-focused vaccination approach was effective in reducing mortality compared to no vaccination, regardless of the RR associated with the seasonal vaccine.  

**Figure:**
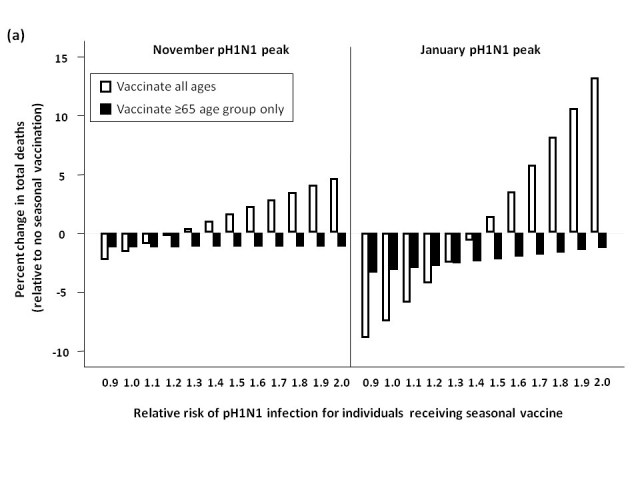

**Figure:**
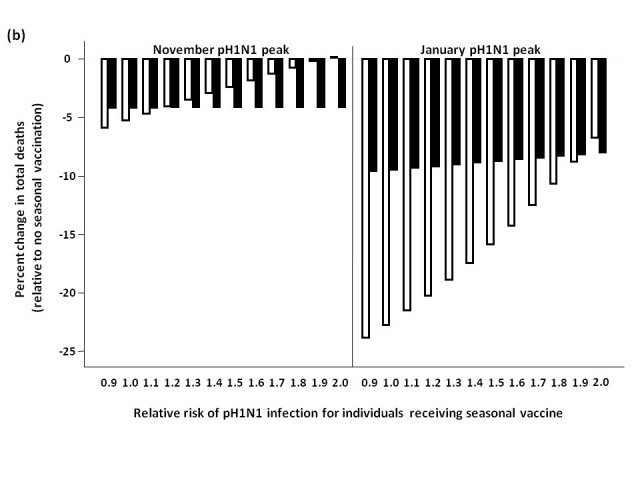
**Figure 4.**  Effect of seasonal vaccine delay on percent change in predicted influenza-attributable mortality, in the presence of (a) low (1.8%) and (b) moderate (6%) levels of circulating seasonal influenza.  The effectiveness of administering seasonal vaccine to all age groups or the ≥65 age group in January was evaluated assuming a pH1N1 epidemic peak in November, 2009 or January, 2010, with pH1N1 vaccination occurring in mid-November.  The percent change in mortality was calculated relative to predicted mortality in the absence of seasonal vaccine administration.  Vaccination coverage for seasonal and pH1N1 influenza is described in Table 1.

### Two-stage age-based vaccination strategy

**Finally, we evaluated the impact of administering seasonal vaccine to the ≥65 age group in October, followed by vaccine distribution to the remaining age groups in January (**Figure 5**).  For a November pH1N1 epidemic peak and low seasonal influenza activity, this approach was not substantively better than vaccinating all age groups in January at lower RR values, and resulted in more total deaths when RR was >1.4, compared to forgoing seasonal immunization completely.  Qualitatively similar results were observed when we assumed a January pH1N1 epidemic peak.  At moderate and high levels of circulating seasonal influenza, the two-stage approach reduced mortality compared to no vaccination.  For a November pH1N1 peak, with moderate levels of seasonal influenza, this strategy was optimal, resulting in the greatest reduction in mortality when the vaccine-associated RR was >1.  

**Figure:**
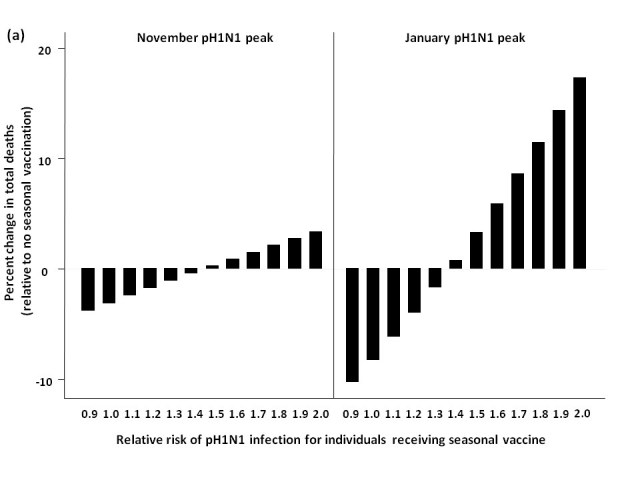

**Figure:**
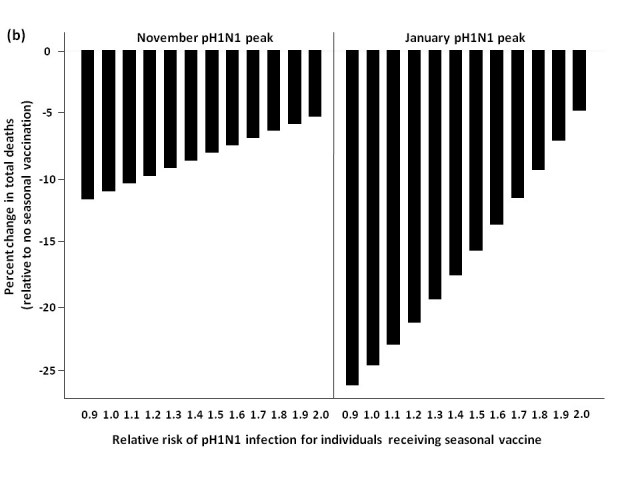
**Figure 5.**  Effect of two-stage vaccination strategy on total predicted influenza-attributable mortality, in the presence of (a) low (1.8%) and (b) moderate (6%) levels of circulating seasonal influenza.  The effectiveness of administering seasonal vaccine to the ≥65 age group in October, followed by the remaining age groups in January was evaluated assuming a pH1N1 epidemic peak in either November, 2009 or January, 2010, with pH1N1 vaccination occurring in mid-November.  Percent increase or decrease in mortality was calculated relative to predicted mortality in the absence of seasonal influenza vaccination.  Vaccination coverage for seasonal and pH1N1 influenza is described in Table 1.

## **Discussion**

We used a mathematical model with two circulating influenza strains to evaluate the effect of modified pH1N1 infection risk following receipt of seasonal influenza vaccine on optimal seasonal vaccination strategies in the Canadian population.  Our projections suggest that, in the presence of the best currently available information on the epidemiology of pH1N1 in Canada, the decisions by several jurisdictional health authorities to restrict or delay the use of seasonal vaccines until after the likely peak of the autumn pandemic wave represents a reasonable choice under uncertainty.  Specifically, in the presence of low levels of co-circulating seasonal influenza strains, even a relatively small enhancement of risk associated with vaccination has a negative impact on total influenza-attributable mortality if the entire population is immunized at usual vaccination coverage.  At higher levels of co-circulating seasonal influenza, the impact of this enhanced risk is less marked, as seasonal vaccine has a proportionately greater role in blunting the impact of mortality associated with seasonal influenza strains.

As countries in the northern hemisphere move into influenza season, it is difficult to predict the amount of circulating seasonal influenza that will be present.  Forecasting the impact of coming influenza seasons has long proved a thorny issue for epidemiologists, even in the absence of an antigenic shift as accompanies this year’s influenza pandemic [21].  However, based on the experience in other countries, including Australia [4] and New Zealand [22], it seems reasonable to assume that pH1N1 will replace H3N2 as the predominant circulating strain.  Although our projections are subject to considerable uncertainty, several clear quantitative patterns emerge that may have application beyond the current year’s influenza pandemic.  First, the magnitude of enhancement of risk by seasonal vaccine is a key driver of the decision to defer seasonal vaccination; with small elevations in risk, early targeted vaccination of older adults, who are most likely to suffer severe sequelae of influenza infection, remains attractive both because of the threat posed to these individuals by seasonal influenza, and also because of the baseline reduced risk of infection in older individuals associated with pH1N1 [9].  At higher levels of risk enhancement by seasonal vaccination, use of seasonal vaccine becomes unattractive, except in the presence of high levels of seasonal influenza.  A second key area of uncertainty relates to the timing of a peak of the second pandemic wave: if (as expected) the wave peaks in autumn (due to the relatively rapid spread of a pandemic strain to which there is little pre-existing immunity in the population) the question of timing or adoption of seasonal vaccination becomes less critical, as risk of infection with the pandemic strain is decreasing sharply as usual seasonal vaccination begins; thus the uncertainty related to enhancement of risk via pH1N1 is much more important if a late pandemic peak is expected.

We note that the findings that motivated this analysis are only now being subjected to scientific scrutiny, and contradict other studies that have found no enhancement of pH1N1 risk [4] or even diminution of pH1N1 risk associated with prior seasonal vaccination against pH1N1 [23].  The Canadian findings could represent artifacts of study design (i.e., be related to residual confounding or bias) or could be due to greater protection against pH1N1 following prior seasonal H1N1 infection than via prior seasonal vaccination.  Under the latter scenario, those who usually refuse seasonal vaccination may have more baseline protection against pH1N1 due to more frequent infection with seasonal H1N1, resulting in a higher level of baseline immunity to infection with the novel virus.  Under any of the scenarios described above, which would be associated with a relative risk of pH1N1 infection of 1 among individuals vaccinated in the 2009/2010 influenza season, our model would project that early seasonal vaccination of the population as a whole would be the recommended vaccination strategy. 

Like any model-based evaluation, our analysis is subject to several important limitations.  As with all mathematical models, this model includes simplifyingassumptions and incorporates parameter values that are subjectto uncertainty, though model calibration to existing data (i.e., assessment of the “fit” between model projections and actual seasonal influenza patterns) was used to validate the model.  Instantaneous administration of vaccine does not reflect the complexity of real-world vaccine programs; however, we would expect this approach to modify the absolute number of deaths observed, but not the relative rank order, compared to the use of a rolling vaccine program in our model.  We did not consider the effect of receipt of seasonal vaccine in previous influenza seasons on enhanced pH1N1 infection risk: if there is a dose-response relationship between seasonal influenza vaccination and pH1N1 infection risk we may have underestimated the impact of seasonal vaccine receipt on pH1N1 risk.  We have made no attempt to evaluate health economic considerations associated with differential vaccination strategies in an atypical influenza season.  Finally, we assumed that there was no modified risk of death among seasonal vaccine recipients in the event that respiratory illness occurred despite infection, though limited evidence suggests that such an effect may exist [24].

In summary, we have developed an age-structured mathematical model of two circulating influenza strains to examine the effect of a seasonal vaccine-associated risk of pH1N1 acquisition on seasonal vaccination strategies during a pandemic.  This model demonstrates that in the presence of uncertainty, withholding, delaying, or targeting seasonal vaccination to older individuals at highest risk from seasonal influenza-related morbidity is reasonable and may result in a net reduction in influenza-related mortality in the coming year. 

## Funding information

AT receives support from the Mathematics of Information Technology and Complex Systems (MITACS) Accelerate Program through both MITACS funding and a matching contribution from the Ontario Agency for Health Protection and Promotion. DF is supported by an Early Researcher Award from the Ontario Ministry of Research and Innovation and is supported by the Canadian Consortium for Pandemic Preparedness Modelling, MITACS and the CIHR.  JK was supported by an Ontario Ministry of Health and Long-Term Care (MOHLTC) Career Scientist Award, a University of Toronto Department of Family and Community Medicine Research Scholar Award, and the Institute for Clinical Evaluative Sciences (ICES). 

The opinions, results and conclusions reported in this paper are those of the authors and are independent from the funding sources. No endorsement by the Institute for Clinical Evaluative Sciences or the Ontario Ministry of Health and Long-Term Care is intended or should be inferred.     

## Competing interests

 DF received matching funds from Sanofi-Pasteur for an Ontario Early Researcher Award on pertussis epidemiology.  Sanofi-Pasteur manufactures a pandemic influenza vaccine not distributed in Canada. No competing interests declared by the other authors.
